# Ethyl 1-(4-chloro­benz­yl)-3-(4-fluoro­phen­yl)-1*H*-pyrazole-5-carboxyl­ate

**DOI:** 10.1107/S1600536811017156

**Published:** 2011-05-11

**Authors:** Yan Qing Ge, Ji Mei Zhang, Guang Liang Wang, Hao Xu, Bo Shi

**Affiliations:** a Taishan Medical College, Tai an, 271016, People’s Republic of China

## Abstract

In the title compound, C_19_H_16_ClFN_2_O_2_, the pyrazole ring makes dihedral angles of 5.15 (6) and 77.72 (6)°, with the fluoro­phenyl and chloro­phenyl rings, respectively.

## Related literature

For the pharmacological activity of pyrazole compounds, see: Ge *et al.* (2007 ▶). For the synthesis of the title compound, see: Li *et al.* (2011 ▶). For the structure of ethyl 1-benzyl-3-(4-fluoro­phen­yl)-1*H*-pyrazole-5-carboxyl­ate, see: Han *et al.* (2011 ▶). For applications of nitro­gen-containing heterocyclic compounds, see: Ge *et al.* (2009 ▶, 2011 ▶).
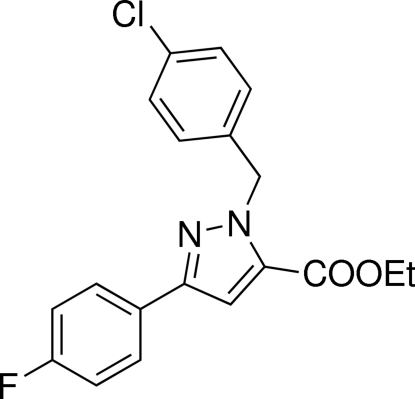


## Experimental

### 

#### Crystal data

C_19_H_16_ClFN_2_O_2_
*M* *_r_* = 358.79
Triclinic, 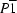

*a* = 8.267 (4) Å
*b* = 10.375 (5) Å
*c* = 11.368 (5) Å
α = 109.128 (7)°
β = 93.269 (7)°
γ = 104.842 (7)°
*V* = 879.8 (7) Å^3^
*Z* = 2
Mo *K*α radiation
μ = 0.24 mm^−1^
*T* = 298 K
0.22 × 0.14 × 0.11 mm

#### Data collection

Bruker SMART APEX CCD diffractometer
Absorption correction: multi-scan (*SADABS*; Bruker, 2005 ▶) *T*_min_ = 0.949, *T*_max_ = 0.974
4589 measured reflections
3091 independent reflections
2570 reflections with *I* > 2σ(*I*)
*R*_int_ = 0.017

#### Refinement

*R*[*F* ^2^ > 2σ(*F* ^2^)] = 0.042
*wR*(*F* ^2^) = 0.117
*S* = 1.05
3091 reflections
227 parameters
H-atom parameters constrained
Δρ_max_ = 0.20 e Å^−3^
Δρ_min_ = −0.32 e Å^−3^

### 

Data collection: *SMART* (Bruker, 2005 ▶); cell refinement: *SAINT* (Bruker, 2005 ▶); data reduction: *SAINT*; program(s) used to solve structure: *SHELXS97* (Sheldrick, 2008 ▶); program(s) used to refine structure: *SHELXL97* (Sheldrick, 2008 ▶); molecular graphics: *XP* in *SHELXTL* (Sheldrick, 2008 ▶); software used to prepare material for publication: *SHELXL97*.

## Supplementary Material

Crystal structure: contains datablocks I, global. DOI: 10.1107/S1600536811017156/fy2010sup1.cif

Structure factors: contains datablocks I. DOI: 10.1107/S1600536811017156/fy2010Isup2.hkl

Additional supplementary materials:  crystallographic information; 3D view; checkCIF report

## Figures and Tables

**Fig. 1.**
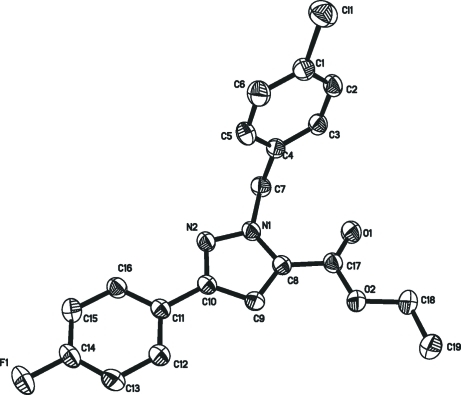
The molecular structure of (I), showing displacement ellipsoids drawn at the 50% probability level.

## supplementary crystallographic information

### Comment

Synthesis of nitrogen-containing heterocyclic compounds has been a subject of great interest due to their applications in the agrochemical and pharmaceutical fields (Ge *et al.*, 2009, 2011). Some pyrazole derivatives which belong to this category have been of interest for their biological activities. Considerable effort has been devoted to the development of novel pyrazole compounds. We report here the crystal structure of the title compound, (I) (Fig. 1)

### Experimental

A mixture of ethyl 3-(4-fluorophenyl)-1*H*-pyrazole-5-carboxylate (0.02 mol), 1-chloro-4-(chloromethyl)benzene (0.0024 mol) and potassium carbonate (0.02 mol) in acetonitrile (100 ml) was heated to reflux for 3 h. The solvent was removed under reduced pressure and the product was isolated by column chromatography on silica gel (yield 88%). Crystals of (I) suitable for X-ray diffraction were obtained by allowing a refluxed solution of the product in ethyl acetate to cool slowly to room temperature and allowing the solvent to evaporate for 1 d.

### Refinement

All H atoms were placed in geometrically calculated positions and refined using a riding model with C—H = 0.97 Å (for CH_2_ groups) and 0.96 Å (for CH_3_ groups), their isotropic displacement parameters were set to 1.2 times (1.5 times for CH_3_ groups) the equivalent displacement parameter of their parent atoms.

### Crystal data

**Table:**

C_19_H_16_ClFN_2_O_2_	*Z* = 2
*M**_r_* = 358.79	*F*(000) = 372
Triclinic, *P*1	*D*_x_ = 1.354 Mg m^−^^3^
Hall symbol: -P 1	Mo *K*α radiation, λ = 0.71073 Å
*a* = 8.267 (4) Å	Cell parameters from 2637 reflections
*b* = 10.375 (5) Å	θ = 2.3–28.0°
*c* = 11.368 (5) Å	µ = 0.24 mm^−^^1^
α = 109.128 (7)°	*T* = 298 K
β = 93.269 (7)°	Block, colourless
γ = 104.842 (7)°	0.22 × 0.14 × 0.11 mm
*V* = 879.8 (7) Å^3^	

### Data collection

**Table:**

Bruker SMART APEX CCD diffractometer	3091 independent reflections
Radiation source: fine-focus sealed tube	2570 reflections with *I* > 2σ(*I*)
graphite	*R*_int_ = 0.017
φ and ω scans	θ_max_ = 25.1°, θ_min_ = 2.2°
Absorption correction: multi-scan (*SADABS*; Bruker, 2005)	*h* = −9→9
*T*_min_ = 0.949, *T*_max_ = 0.974	*k* = −7→12
4589 measured reflections	*l* = −13→13

### Refinement

**Table:**

Refinement on *F*^2^	Secondary atom site location: difference Fourier map
Least-squares matrix: full	Hydrogen site location: inferred from neighbouring sites
*R*[*F*^2^ > 2σ(*F*^2^)] = 0.042	H-atom parameters constrained
*wR*(*F*^2^) = 0.117	*w* = 1/[σ^2^(*F*_o_^2^) + (0.0578*P*)^2^ + 0.1997*P*] where *P* = (*F*_o_^2^ + 2*F*_c_^2^)/3
*S* = 1.05	(Δ/σ)_max_ < 0.001
3091 reflections	Δρ_max_ = 0.20 e Å^−^^3^
227 parameters	Δρ_min_ = −0.32 e Å^−^^3^
0 restraints	Extinction correction: *SHELXL*, Fc^*^=kFc[1+0.001xFc^2^λ^3^/sin(2θ)]^-1/4^
Primary atom site location: structure-invariant direct methods	Extinction coefficient: 0.377 (15)

### Special details

**Table:**

Geometry. All e.s.d.'s (except the e.s.d. in the dihedral angle between two l.s. planes) are estimated using the full covariance matrix. The cell e.s.d.'s are taken into account individually in the estimation of e.s.d.'s in distances, angles and torsion angles; correlations between e.s.d.'s in cell parameters are only used when they are defined by crystal symmetry. An approximate (isotropic) treatment of cell e.s.d.'s is used for estimating e.s.d.'s involving l.s. planes.
Refinement. Refinement of *F*^2^ against ALL reflections. The weighted *R*-factor *wR* and goodness of fit *S* are based on *F*^2^, conventional *R*-factors *R* are based on *F*, with *F* set to zero for negative *F*^2^. The threshold expression of *F*^2^ > σ(*F*^2^) is used only for calculating *R*-factors(gt) *etc*. and is not relevant to the choice of reflections for refinement. *R*-factors based on *F*^2^ are statistically about twice as large as those based on *F*, and *R*- factors based on ALL data will be even larger.

### Fractional atomic coordinates and isotropic or equivalent isotropic displacement parameters (Å^2^)

**Table:**

	*x*	*y*	*z*	*U*_iso_*/*U*_eq_	
Cl1	1.50553 (8)	0.97753 (7)	0.28534 (7)	0.0902 (3)	
F1	0.58679 (18)	0.70217 (16)	1.03659 (11)	0.0901 (4)	
O1	0.8333 (2)	0.38223 (15)	0.13974 (12)	0.0730 (4)	
O2	0.85036 (17)	0.24464 (14)	0.25387 (12)	0.0635 (4)	
N1	0.76250 (18)	0.58191 (16)	0.36486 (13)	0.0520 (4)	
N2	0.72669 (19)	0.64677 (16)	0.47978 (13)	0.0538 (4)	
C1	1.2960 (3)	0.8828 (2)	0.28289 (19)	0.0623 (5)	
C2	1.2032 (3)	0.7838 (2)	0.17127 (17)	0.0613 (5)	
H2	1.2510	0.7663	0.0976	0.074*	
C3	1.0381 (3)	0.7106 (2)	0.17021 (16)	0.0581 (5)	
H3	0.9750	0.6430	0.0949	0.070*	
C4	0.9639 (2)	0.73540 (19)	0.27895 (15)	0.0530 (4)	
C5	1.0613 (3)	0.8359 (2)	0.39014 (17)	0.0665 (5)	
H5	1.0142	0.8537	0.4641	0.080*	
C6	1.2269 (3)	0.9101 (2)	0.39306 (19)	0.0735 (6)	
H6	1.2910	0.9774	0.4681	0.088*	
C7	0.7810 (2)	0.6583 (2)	0.27612 (17)	0.0589 (5)	
H7A	0.7168	0.7269	0.2968	0.071*	
H7B	0.7342	0.5910	0.1916	0.071*	
C8	0.7893 (2)	0.45469 (18)	0.35425 (15)	0.0489 (4)	
C9	0.7673 (2)	0.43606 (18)	0.46724 (15)	0.0502 (4)	
H9	0.7764	0.3590	0.4890	0.060*	
C10	0.7285 (2)	0.55756 (18)	0.54247 (15)	0.0480 (4)	
C11	0.6912 (2)	0.59545 (18)	0.67292 (15)	0.0490 (4)	
C12	0.7055 (2)	0.5107 (2)	0.74363 (17)	0.0599 (5)	
H12	0.7390	0.4291	0.7082	0.072*	
C13	0.6705 (3)	0.5465 (2)	0.86630 (18)	0.0663 (5)	
H13	0.6798	0.4896	0.9133	0.080*	
C14	0.6221 (2)	0.6670 (2)	0.91642 (17)	0.0633 (5)	
C15	0.6065 (3)	0.7534 (2)	0.85037 (18)	0.0642 (5)	
H15	0.5733	0.8349	0.8870	0.077*	
C16	0.6412 (2)	0.7171 (2)	0.72777 (17)	0.0564 (4)	
H16	0.6309	0.7747	0.6817	0.068*	
C17	0.8260 (2)	0.35999 (19)	0.23761 (16)	0.0535 (4)	
C18	0.8920 (3)	0.1438 (2)	0.1458 (2)	0.0723 (6)	
H18A	0.9889	0.1920	0.1161	0.087*	
H18B	0.7970	0.1014	0.0776	0.087*	
C19	0.9320 (3)	0.0317 (3)	0.1868 (3)	0.0882 (7)	
H19A	1.0243	0.0751	0.2555	0.132*	
H19B	0.9631	−0.0351	0.1177	0.132*	
H19C	0.8343	−0.0171	0.2136	0.132*	

### Atomic displacement parameters (Å^2^)

**Table:**

	*U*^11^	*U*^22^	*U*^33^	*U*^12^	*U*^13^	*U*^23^
Cl1	0.0733 (4)	0.0979 (5)	0.1037 (5)	0.0228 (3)	0.0231 (3)	0.0415 (4)
F1	0.1132 (10)	0.1118 (10)	0.0493 (7)	0.0361 (8)	0.0303 (6)	0.0283 (7)
O1	0.1059 (11)	0.0717 (9)	0.0482 (8)	0.0343 (8)	0.0217 (7)	0.0219 (7)
O2	0.0808 (9)	0.0592 (8)	0.0567 (8)	0.0294 (7)	0.0222 (6)	0.0198 (6)
N1	0.0610 (9)	0.0581 (9)	0.0442 (8)	0.0220 (7)	0.0137 (6)	0.0229 (7)
N2	0.0632 (9)	0.0583 (9)	0.0459 (8)	0.0234 (7)	0.0151 (6)	0.0206 (7)
C1	0.0701 (12)	0.0634 (11)	0.0642 (12)	0.0276 (9)	0.0183 (9)	0.0292 (10)
C2	0.0821 (13)	0.0669 (12)	0.0503 (10)	0.0354 (10)	0.0256 (9)	0.0277 (9)
C3	0.0814 (13)	0.0575 (10)	0.0414 (9)	0.0275 (9)	0.0134 (8)	0.0192 (8)
C4	0.0727 (11)	0.0539 (10)	0.0438 (9)	0.0274 (9)	0.0146 (8)	0.0240 (8)
C5	0.0851 (14)	0.0716 (13)	0.0418 (10)	0.0225 (11)	0.0198 (9)	0.0173 (9)
C6	0.0840 (15)	0.0748 (14)	0.0507 (11)	0.0163 (11)	0.0079 (10)	0.0139 (10)
C7	0.0733 (12)	0.0672 (11)	0.0492 (10)	0.0293 (9)	0.0124 (8)	0.0300 (9)
C8	0.0494 (9)	0.0522 (9)	0.0449 (9)	0.0146 (7)	0.0074 (7)	0.0171 (7)
C9	0.0546 (10)	0.0512 (9)	0.0475 (9)	0.0162 (8)	0.0088 (7)	0.0202 (8)
C10	0.0473 (9)	0.0538 (10)	0.0433 (9)	0.0134 (7)	0.0071 (7)	0.0188 (7)
C11	0.0463 (9)	0.0546 (10)	0.0445 (9)	0.0117 (7)	0.0068 (7)	0.0180 (8)
C12	0.0726 (12)	0.0609 (11)	0.0502 (10)	0.0217 (9)	0.0130 (8)	0.0225 (9)
C13	0.0807 (13)	0.0731 (13)	0.0521 (11)	0.0215 (11)	0.0138 (9)	0.0315 (10)
C14	0.0646 (11)	0.0799 (13)	0.0419 (9)	0.0145 (10)	0.0134 (8)	0.0212 (9)
C15	0.0692 (12)	0.0685 (12)	0.0543 (11)	0.0262 (10)	0.0157 (9)	0.0152 (9)
C16	0.0624 (11)	0.0623 (11)	0.0501 (10)	0.0222 (9)	0.0123 (8)	0.0236 (8)
C17	0.0549 (10)	0.0546 (10)	0.0478 (10)	0.0135 (8)	0.0097 (7)	0.0157 (8)
C18	0.0880 (14)	0.0623 (12)	0.0657 (12)	0.0312 (11)	0.0238 (10)	0.0125 (10)
C19	0.0947 (17)	0.0722 (14)	0.112 (2)	0.0396 (13)	0.0408 (14)	0.0357 (14)

### Geometric parameters (Å, °)

**Table:**

Cl1—C1	1.753 (2)	C8—C9	1.375 (2)
F1—C14	1.363 (2)	C8—C17	1.470 (2)
O1—C17	1.210 (2)	C9—C10	1.398 (2)
O2—C17	1.331 (2)	C9—H9	0.9300
O2—C18	1.454 (2)	C10—C11	1.477 (2)
N1—N2	1.348 (2)	C11—C16	1.390 (3)
N1—C8	1.362 (2)	C11—C12	1.392 (2)
N1—C7	1.466 (2)	C12—C13	1.388 (3)
N2—C10	1.343 (2)	C12—H12	0.9300
C1—C2	1.373 (3)	C13—C14	1.366 (3)
C1—C6	1.381 (3)	C13—H13	0.9300
C2—C3	1.379 (3)	C14—C15	1.368 (3)
C2—H2	0.9300	C15—C16	1.387 (3)
C3—C4	1.389 (2)	C15—H15	0.9300
C3—H3	0.9300	C16—H16	0.9300
C4—C5	1.387 (3)	C18—C19	1.488 (3)
C4—C7	1.512 (3)	C18—H18A	0.9700
C5—C6	1.380 (3)	C18—H18B	0.9700
C5—H5	0.9300	C19—H19A	0.9600
C6—H6	0.9300	C19—H19B	0.9600
C7—H7A	0.9700	C19—H19C	0.9600
C7—H7B	0.9700		
			
C17—O2—C18	115.92 (15)	N2—C10—C11	119.71 (15)
N2—N1—C8	111.63 (13)	C9—C10—C11	129.52 (15)
N2—N1—C7	118.43 (15)	C16—C11—C12	118.60 (17)
C8—N1—C7	129.72 (15)	C16—C11—C10	120.50 (16)
C10—N2—N1	105.41 (14)	C12—C11—C10	120.90 (16)
C2—C1—C6	121.27 (19)	C13—C12—C11	120.85 (19)
C2—C1—Cl1	119.37 (15)	C13—C12—H12	119.6
C6—C1—Cl1	119.37 (17)	C11—C12—H12	119.6
C1—C2—C3	118.90 (17)	C14—C13—C12	118.54 (18)
C1—C2—H2	120.6	C14—C13—H13	120.7
C3—C2—H2	120.6	C12—C13—H13	120.7
C2—C3—C4	121.55 (17)	F1—C14—C13	118.73 (19)
C2—C3—H3	119.2	F1—C14—C15	118.72 (19)
C4—C3—H3	119.2	C13—C14—C15	122.55 (18)
C5—C4—C3	118.05 (18)	C14—C15—C16	118.70 (19)
C5—C4—C7	120.71 (16)	C14—C15—H15	120.6
C3—C4—C7	121.22 (16)	C16—C15—H15	120.6
C6—C5—C4	121.21 (18)	C15—C16—C11	120.75 (18)
C6—C5—H5	119.4	C15—C16—H16	119.6
C4—C5—H5	119.4	C11—C16—H16	119.6
C5—C6—C1	119.02 (19)	O1—C17—O2	124.03 (17)
C5—C6—H6	120.5	O1—C17—C8	125.40 (18)
C1—C6—H6	120.5	O2—C17—C8	110.57 (15)
N1—C7—C4	112.17 (14)	O2—C18—C19	107.60 (18)
N1—C7—H7A	109.2	O2—C18—H18A	110.2
C4—C7—H7A	109.2	C19—C18—H18A	110.2
N1—C7—H7B	109.2	O2—C18—H18B	110.2
C4—C7—H7B	109.2	C19—C18—H18B	110.2
H7A—C7—H7B	107.9	H18A—C18—H18B	108.5
N1—C8—C9	106.76 (15)	C18—C19—H19A	109.5
N1—C8—C17	122.96 (15)	C18—C19—H19B	109.5
C9—C8—C17	130.22 (17)	H19A—C19—H19B	109.5
C8—C9—C10	105.43 (15)	C18—C19—H19C	109.5
C8—C9—H9	127.3	H19A—C19—H19C	109.5
C10—C9—H9	127.3	H19B—C19—H19C	109.5
N2—C10—C9	110.77 (15)		
			
C8—N1—N2—C10	−0.88 (18)	C8—C9—C10—N2	−0.08 (19)
C7—N1—N2—C10	−175.99 (14)	C8—C9—C10—C11	179.54 (16)
C6—C1—C2—C3	−0.1 (3)	N2—C10—C11—C16	5.1 (2)
Cl1—C1—C2—C3	−179.55 (14)	C9—C10—C11—C16	−174.51 (17)
C1—C2—C3—C4	0.3 (3)	N2—C10—C11—C12	−174.77 (16)
C2—C3—C4—C5	−0.5 (3)	C9—C10—C11—C12	5.6 (3)
C2—C3—C4—C7	177.88 (16)	C16—C11—C12—C13	0.1 (3)
C3—C4—C5—C6	0.4 (3)	C10—C11—C12—C13	179.94 (17)
C7—C4—C5—C6	−177.99 (18)	C11—C12—C13—C14	−0.2 (3)
C4—C5—C6—C1	−0.1 (3)	C12—C13—C14—F1	179.67 (17)
C2—C1—C6—C5	0.0 (3)	C12—C13—C14—C15	0.2 (3)
Cl1—C1—C6—C5	179.45 (16)	F1—C14—C15—C16	−179.51 (17)
N2—N1—C7—C4	95.64 (18)	C13—C14—C15—C16	0.0 (3)
C8—N1—C7—C4	−78.4 (2)	C14—C15—C16—C11	−0.1 (3)
C5—C4—C7—N1	−57.5 (2)	C12—C11—C16—C15	0.1 (3)
C3—C4—C7—N1	124.17 (18)	C10—C11—C16—C15	−179.79 (16)
N2—N1—C8—C9	0.85 (19)	C18—O2—C17—O1	1.5 (3)
C7—N1—C8—C9	175.25 (16)	C18—O2—C17—C8	−178.52 (16)
N2—N1—C8—C17	178.25 (15)	N1—C8—C17—O1	−1.4 (3)
C7—N1—C8—C17	−7.3 (3)	C9—C8—C17—O1	175.33 (19)
N1—C8—C9—C10	−0.45 (18)	N1—C8—C17—O2	178.63 (15)
C17—C8—C9—C10	−177.60 (17)	C9—C8—C17—O2	−4.6 (3)
N1—N2—C10—C9	0.57 (18)	C17—O2—C18—C19	174.11 (17)
N1—N2—C10—C11	−179.09 (14)		
